# IT'S ABOUT WHO YOU KNOW: RELATIONSHIPS BETWEEN SOCIAL NETWORKS, COGNITIVE FUNCTION, AND BRAIN HEALTH IN LATER LIFE

**DOI:** 10.1093/geroni/igac059.949

**Published:** 2022-12-20

**Authors:** Lucas Hamilton, Sara Moorman

## Abstract

Evidence has been steadily increasing about the benefit of social connectedness for healthy aging. Yet, thanks to the complexity of human social interactions, it remains difficult to pinpoint specific benefits and mechanisms by which such benefits emerge. We offer this symposium to address this issue by presenting new findings from ongoing work at Indiana University in collaboration with the Indiana Alzheimer’s Disease Research Center. First, an overview of recent literature will be presented alongside a brief introduction to egocentric network analysis and its utility in probing social determinants of health. Next, we turn our attention to characteristics of social networks and their importance for cognitive health in later life. Different types of social enrichment will be evaluated in terms of their respective influence on cognitive reserve (i.e., neuropsychological evaluations, brain health). Additional attention will be paid to the comparison of emotionally supportive (i.e., social bonding) and more informationally supportive (i.e., social bridging) ties as promoters of brain health. Then, the final talks will describe potential avenues for increasing social connectedness in older adulthood. Individual differences in social cognitive function, namely Theory of Mind (i.e., reading another person’s thoughts, feelings, and intentions), has been implicated as mechanism by which older adults can maintain larger networks with more weak ties, in turn, promoting cognitive function. Separately, the presence of ambivalent ties (i.e., connections that beget positivity and negativity) may serve as a window of opportunity whereby older adults can leverage strengths in emotion regulation to maintain these ties without negative health consequences.

